# Size matters: Anaerobic granules exhibit distinct ecological and physico-chemical gradients across biofilm size

**DOI:** 10.1016/j.ese.2025.100561

**Published:** 2025-03-27

**Authors:** Anna Trego, Cristina Morabito, Isabelle Bourven, Giles Guibaud, Vincent O'Flaherty, Gavin Collins, Umer Zeeshan Ijaz

**Affiliations:** a Sustainable World Section, School of Biological and Chemical Sciences, University of Ireland Galway, University Road, Galway, H91 TK33, Ireland; b E2lim, UR 24 133, Université de Limoges, Faculté des Sciences Techniques, 123 Avenue Albert Thomas, 87060 Limoges Cedex, France; c Mazumdar-Shaw Advanced Research Centre (ARC), University of Glasgow, 11 Chapel Lane, Western Site, Glasgow, G11 6EW, United Kingdom

**Keywords:** Anaerobic digestion, Granules, Biofilms, Methanogenesis, Size

## Abstract

Anaerobic biological decomposition of organic matter is ubiquitous in Nature wherever anaerobic environments prevail, and is catalysed by hydrolytic, fermentative, acetogenic, methanogenic, and various other groups. It is also harnessed in innovative ways in engineered systems that may rely on small (0.1–4.0 mm), spherical, anaerobic granules. These biofilms are crucial to the operational success of a range of widely applied engineered-ecosystems designed for wastewater treatment. The structure and function of granule microbiomes underpin their utility. Here, granules were separated into ten size fractions (proxies for age), hypothesizing that small granules are ‘young’ and larger ones are ‘old’. Gradients were observed across size in terms of volatile solids, density, settleability, biofilm morphology, methanogenic activity, and profiles of extracellular polymeric substances, suggesting ongoing development of physico-chemical characteristics as granules develop. Short-read amplicon sequencing indicated a negative relationship between granule size and community diversity. Furthermore, as size increased, the methanogenic archaea dominated the microbiome. Small granules were found to harbour a sub-group of highly specific taxa, and the identification of generalists and specialists may point to substantial resilience of the microbiome. The findings of this study indicate opportunities for precision management of wastewater treatment systems. They suggest that size is an important indicator for aggregate utility – size may, indeed, determine many of the characteristics of both the individual-granule microbiomes and the overall function of a wastewater treatment system.

## Highlights

• Size-separated anaerobic granules exhibit gradients in physico-chemical parameters.
• Microbial community diversity decreases with increasing size.
• Small granules contain niche-specific taxa; large granules are cosmopolitan.
• Large granules are dominated by methanogenic archaea.
• Medium-sized granules may be the most optimal.

## Graphical abstract

**Figure:**
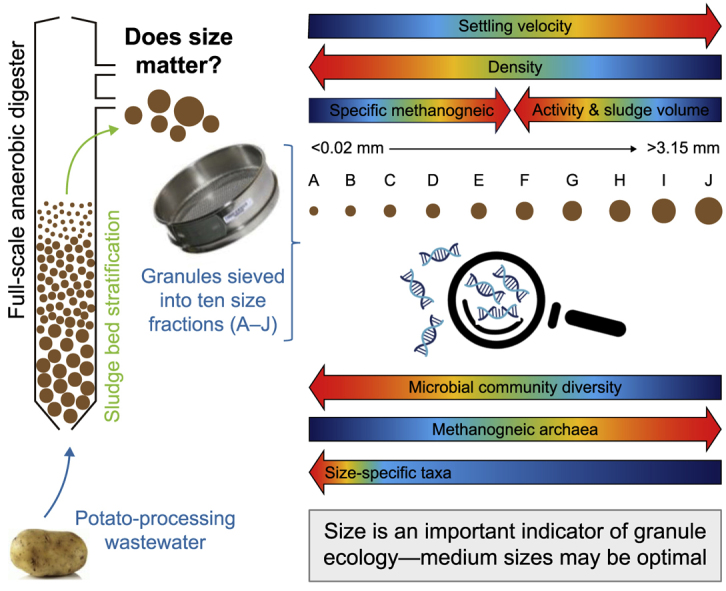

## 1 Introduction

Evidence suggests, time and again, that microbiomes play commanding roles in whole-ecosystem dynamics [1]. Global carbon cycling comprises (i) primary production by photo- and chemo-synthesis and (ii) carbon consumption by respiration, but also relies fundamentally on the anaerobic, biological decomposition of organic matter wherever suitable environments prevail, such as in saturated wetlands, coastal, lake, and marine sediments; and ruminant and termite guts, among many others [[2], [3], [4]]. Indeed, it is estimated that anaerobic microbial activity from these environments releases ∼1 billion tons of global methane (CH_4_) per year [5,6], representing a significant global methane source [7,8]. This natural process, called anaerobic digestion (AD), is mediated by the collective, sequential, and cooperative action of several trophic groups of microorganisms, including hydrolytic bacteria, fermenters, organic-acid-oxidizers, and—finally—the evolutionarily ancient, methanogenic archaea feeding on a narrow range of substrates including acetate, methanol and H_2_/CO_2_. Methanogenic consortia of this type comprise complex microbial communities of bacteria and archaea and may include a range of syntrophic species operating within extremely narrow thermodynamic profitability windows [9].

The AD process is widely harnessed for wastewater treatment in engineered digester systems [[10], [11], [12]]. Anaerobic granules are roughly spherical biofilm aggregates, approximately 0.1–5.0 mm in diameter, applied in up-flow digesters. Each granule comprises a diverse microbial consortium and harbours the microbial community required for complete feedstock mineralisation [13]. The granular structure supports efficient substrate transfer between trophic groups and protection from toxins and perturbations [14]. In a pivotal review, Hulshoff Pol et al. (2004) [15] proposed several theories based on ecological, physico-chemical characteristics, and thermodynamic principles to explain the granulation process. Most agree that inert particles likely play a critical role as substrata in granule formation and that the early stages of granule development likely mimic the classical models of cell attachment and biofilm formation on solid surfaces [16]. Most also agree that acetoclastic methanogens from the *Methanothrix* (*Methanosaeta*) genus are key to granulation, providing a core of tangled filaments around which other cells aggregate [17]. Several studies focused on the early stages of biofilm development, but very few studied the growth and maturation of granules and the microbiome assembly over time [15].

Indeed, many studies have described aggregate formation from diverse microbial communities, such as the bacterial colonisation and organisation of marine particulate organic matter [18,19]. Nonetheless, the mechanisms by which complex communities assemble to form stable biofilms are still only poorly understood. Others describe interactions between neutral and species-sorting processes and the roles of generalists and specialists in community assembly [[20], [21], [22]].

Whilst full-scale anaerobic digesters (with volumes typically up to several hundred cubic metres) contain millions of individual granules, not all granules are the same. Ahn (2000) [23] found the typical range of granule diameters to be 0.1–5.0 mm, and in a characterization by Diaz et al. (2006) [24] different granule morphologies were observed from single digesters. They separated granules by colour and observed differences in granule size and structure. Our recent research has indicated that granules within distinct size ranges harbour statistically identical microbiomes [13], even though the community structure in granules of different sizes may differ [25]. Each granule, therefore, represents a perfectly parameterised whole biofilm ecosystem. Consequently, and rather uniquely within both natural and constructed environments, anaerobic granules may be characterised as distinct and fully replicated whole microbial communities [13].

Now, we hypothesise that differently sized granules represent different stages of biofilm development and that granules taken from a single digester at a single point in time, having survived the same environmental conditions, may represent different stages of growth where the smallest granules are at the earliest stages of formation, and the largest granules are the oldest and most mature. This assumption is supported by the current understanding of granule formation [14,15,26], how other types of biofilms grow and spread [27], and our previous work studying granule growth [17,28]. Therefore, we intensively characterised anaerobic granules from a full-scale digester across multiple discrete size fractions to characterise morphological, physico-chemical, physiological, and ecological differences across a set of highly-resolved granule size fractions. We hypothesised that (i) there would be observable differences in biofilm characteristics and microbial community structure across granule sizes, and (ii) it would be possible to establish a correlation between these differences such that granule size may ultimately serve as an indicator of both ecological ‘fitness’ and bioreactor process performance. This provides an interesting new perspective concerning biofilm development in a controlled system.

## 2 Materials and methods

### 2.1 Source of biomass and size fractionation

Anaerobic granular biomass was obtained from a full-scale, mesophilic (37 °C) upflow anaerobic sludge bed (UASB) digester treating potato-processing wastewater in the Netherlands. Biomass was size-separated into ten distinct size fractions (A–J) based on granule diameter, with Fraction A representing the smallest granules (diameter, <0.20 mm) and Fraction J containing the largest granules (diameter, 3.15–4.00 mm; Table 1). The size range was determined by passing the granules through a series of stainless-steel grading sieves (Fisher Scientific, Geel, Belgium). The sieves were stacked according to size, with the largest aperture (3.15 mm) on top and the smallest on the bottom. To determine granule fraction, granules were allowed to pass through the sieves (while being washed with tap water) until they were caught by the sieve mesh. Each fraction was then separately transferred to 1-L graduated bottles (Fisher Scientific, Geel, Belgium), suspended in 1x phosphate-buffered saline (PBS; Fisher Scientific, Geel, Belgium), sealed with a rubber stopper, sparged with N_2_ gas, and allowed to settle for 1 h before determining the settled volume.

**Table 1:** Size (diameter) of fractions A–J.

Fraction	Size range (mm)
A	<0.20
B	0.20–0.40
C	0.40–0.60
D	0.60–0.80
E	0.80–1.00
F	1.00–1.40
G	1.40–1.80
H	1.80–2.00
I	2.00–3.15
J	3.15–4.00

### 2.2 Total and volatile solids and granule settling velocity and density

The total solids (TS) and volatile solids (VS) concentrations of granules from each size fraction were determined using the standard loss-on-ignition technique [29]. We determined the settling velocity and density of granules (*n* = 10) from each size fraction (A–J). A 1-m long, clear, acrylic tube, fitted with a stopper at one end, was fastened vertically and filled with deionised water. Two markings were made on the outside of the tube, at 0.3 m and 0.6 m from the top, and the water temperature was recorded. The diameter of individual granules was measured using electronic digital calipers. Granules were individually dropped into the water column, and the time (seconds) required for each granule to travel 0.3 m (the distance between the two markings) was measured. The settling velocity was the distance (0.3 m) divided by the settling time. We applied Stokes’ Law to determine granule density.

### 2.3 Scanning electron microscopy

Granules (*n* = 3) from each size fraction were randomly selected for scanning electron microscopy (SEM) imaging. Granules were placed in clean, individual 1.5-mL microcentrifuge tubes and covered with 2.5 % (w/v) glutaraldehyde in 0.5 M cacodylate buffer (pH 7.2). The tubes were inverted gently and incubated overnight at 4 °C. The supernatant was removed, and granules were washed three times in 1X PBS before being dehydrated by passing through a series of 10-min ethanol washes using 50 %, 70 % and 90 % ethanol. Dehydrated granules were placed on carbon tabs, which were then fastened to aluminium stubs. An aliquot of 25 μL hexamethyldisilazane (HMDS) was placed under a fume hood on each granule and dried overnight. Specimens were gold-sputtered and imaged in a scanning electron microscope (Hitachi S-2600, Mountain View, California, USA).

### 2.4 Extraction and characterization of extracellular polymeric substances

Loosely bound (LB) and tightly bound (TB) extracellular polymeric substances (EPS) was extracted in duplicate from sludge from each size fraction using the cation exchange resin (CER) technique [30,31]. Colorimetric assays were used to investigate the biochemical composition of the EPS using a spectral photometer (Cadas 50 S, Dr Lange, Berlin, Germany). Concentrations of proteins and humic-like substances (HLS) were determined and corrected [32,33]. Bovine serum albumin (96 %, Sigma-Aldrich, St. Louis, Missouri, USA) was used as a standard for proteins, and humic acids (Sigma-Aldrich, St. Louis, Missouri, USA) as the standard for HLS. Following [34], polysaccharides were measured following a glucose standard.

### 2.5 Specific methanogenic activity

A specific methanogenic activity (SMA) buffer solution was prepared in a round-bottom flask by combining 0.4 mL 0.0001 % (w/v) resazurin (Fisher Scientific, Geel, Belgium), 0.56 g cysteine hydrochloride monohydrate and enough distilled water (dH_2_O) to bring the volume to 700 mL. The pH was adjusted to 7.0–7.1 by dropwise addition of 8 M NaOH, and the final volume was adjusted to 1 L. The solution was boiled until clear, immediately sealed, and cooled on ice with constant N_2_ sparging until at 50 °C when 3.05 g sodium bicarbonate was added before sealing the flask. The SMA buffer was added with sludge granules, in triplicate for each size fraction, to 60-mL glass bottles to give a final volume of buffer and granules of 10 mL and a final VS concentration of 4 g L^−1^. The bottles were sealed and N_2_-flushed before acclimatisation at 37 °C for 48 h. Aliquots of 0.1 mL soluble substrates were added to separate respective bottles to give final concentrations of 30 mM acetate, 15 mM butyrate or 30 mM propionate. No substrate controls measured background activity. H_2_/CO_2_ (80:20, v/v) was added at 1 bar for 20 s to test for autotrophic methanogenesis. N_2_/CO_2_ (80:20, v/v) was used to control H_2_-fed assays. Headspace biogas pressure was measured as millivolts (mv) using a handheld pressure transducer (CentrePoint Electronics, Galway, Ireland), and converted to biogas volume (mL) using a headspace correction factor [35,36]. Gas chromatography (CP-3800, VARIAN Inc., Walnut Creek, California, USA) was used to determine the methane concentration (%) in the biogas, and the accumulation rate was plotted. As before, the precise, *in situ* concentration of VS in each bottle was determined by drying and burning. In the case of soluble substrates, SMAs were determined under STP conditions as the daily rate of methane production as a function of the total VS. SMA for gaseous substrates were calculated using a similar approach; however, the rate was computed using the reaction stoichiometry of 4:1 M of H_2_ consumption to methane production.

### 2.6 DNA extraction

A mass of 0.1 g wet granules from each size fraction was weighed into respective, triplicate sterile tubes. DNA was extracted on ice following the DNA/RNA co-extraction method described by [NO_PRINTED_FORM] [37], which is based on bead beating in 5 % (w/v) cetyl trimethylammonium bromide (CTAB) extraction buffer, followed by phenol-chloroform extraction. The integrity of nucleic acids was assessed using a nanodrop (Thermo Fisher Scientific, Waltham, MA, USA). Concentrations were determined using a Qubit fluorometer (Invitrogen, Carlsbad, California, USA) and normalized to 5 ng DNA μL^−1^ before storage at −80 °C.

### 2.7 DNA sequencing, bioinformatics, and statistical analysis

Amplification of the 16S rRNA gene sequences was performed by The Foundation for the Promotion of Health and Biomedical Research of Valencia Region, FISABIO (Valencia, Spain) using the universal bacterial and archaeal primer set: forward primer 515F and reverse primer 806R [38]. The resulting amplicon library of short inserts was sequenced on the Illumina MiSeq platform. Abundance tables were generated by constructing amplicon sequencing variants (ASVs) using the qiime2 pipeline and the DADA2 algorithm [39]. For ensuing statistical analysis, the reads were rarefied to the sample with the minimum number of sample reads. This yielded a 30 (sample) x 3041 (ASV) abundance table. Statistical analyses were performed in R (v3.4.4) using the combined data generated from the bioinformatics and meta data associated with the study. Statistical analyses used in this study included diversity analyses, ensemble quotient optimisation (EQO), specificity, and Levin's niche-breadth analysis. Details of the bioinformatics and statistics are described further in Supplementary Materials.

### 2.8 Data availability

The amplicon sequencing data from this study are available on the European Nucleotide Archive under the study accession number PRJEB28212 (http://www.ebi.ac.uk/ena/data/view/PRJEB28212).

## 3 Results

We categorised granules into ten distinct size fractions (A–J) to better understand how granule properties relate to their communities, with A representing the smallest and J the largest. We performed a series of physico-chemical analyses, including (i) analysis of the overall size distribution, (ii) measurements of total and volatile solids (TS and VS), (iii) granule density and settling velocity, (iv) ultrastructure morphology (using SEM), (v) EPS profiling, and (vi) rates of methane production using SMA assays. Data describing community structure and dynamics are from short-read amplicon sequencing (three replicate samples from each size fraction: *n* = 30 total samples). We sequenced samples from each fraction, which included multiple individual granules; single granules were not analysed in this study.

### 3.1 Gradients in physico-chemical properties across granule sizes

**Fig. 1:**
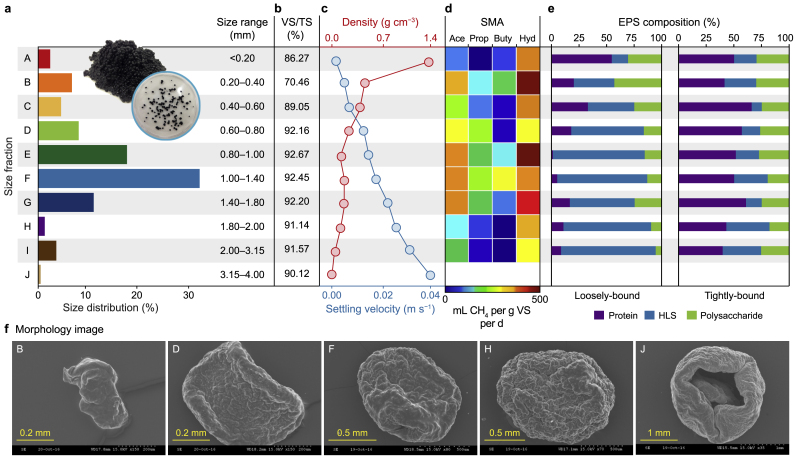
Physico-chemical and physiological data from granule size fractions (A–J). **a**, Bar plot indicating the size ranges of respective fractions, along with relative volumetric contributions to the sludge. **b**, Volatile solids (VS) proportions of total solids (TS). **c**, Scatter plot illustrating the settling velocity of granules (*n* = 10) from each size fraction. **d**, Heatmap depicting specific methanogenic activity (SMA) of sludge samples (*n* = 3) from each size fraction (except fraction J due to the low number of granules of that size) against acetate (Ace), propionate (Prop), butyrate (Buty), and H_2_/CO_2_ (Hyd). **e**, Stacked bar charts showing relative concentrations of proteins, humic-like substances (HLS), and polysaccharides components in loosely-bound and tightly-bound-extracellular polymeric substances (EPS) extracted from each size fraction (except fraction J). **f,** Typical scanning electron microscopy (SEM) micrographs of selected granules (from fractions B, D, F, H, and J). See Supplementary Materials Tables S1–S3 for exact values on all physico-chemical data and Supplementary Material Fig. S1 for SEM images of all fractions and magnifications.

A normal distribution of granule sizes was observed, with a majority (>75 %) of the sludge volume comprising medium-range granules (fractions D–G; Fig. 1a; Supplementary Material Table S1). No granules >4 mm were found. The VS proportion of TS was relatively high (average, 91.8 %; σ^2^, 0.88) in granules from medium and larger fractions (D–J) but lower in smaller granules (86.2 %, 70.4 % and 89.0 % in fractions A, B and C, respectively; Fig. 1b). A trajectory in granule ultrastructure was observed across the size fractions (Fig. 1c; Supplementary Material Fig. S1). The smallest granules (Fractions A–C) presented as ‘flakes’; medium-sized granules (Fractions D–F) appeared better defined but were ‘flat’ (i.e. not spherical); larger granules (Fractions G–I) were distinctly spherical and more ‘granular’; and in the largest granules (Fraction J), large cracks and void spaces were observed, and the granules appeared to have broken apart, losing structural integrity (Fig. 1c). The density and settling velocity of granules exhibited a broadly linear relationship across the size fractions (Fig. 1d); however, they were inversely correlated: smaller granules (Fraction A) had high densities (104.6 g cm^−3^) but low settling velocities (average, 0.001 m s^−1^), whilst large granules (Fraction J) were less dense (6.82 g cm^−3^) and settled faster (average, 0.041 m s^−1^) due to the increased surface area:volume ratio. There was no change in the composition of tightly-bound (TB)-EPS across the size fractions—the proportion of each of the three examined components (protein, 53.2 % (σ^2^, 7.1); humic-like substances (HLS), 23.0 % (σ^2^, 7.5); and polysaccharides, the remaining 23.7 % (σ^2^, 2.8)) was relatively stable. However, a gradient was observed across the size fractions in the proportions of loosely-bound (LB)-EPS components; for example, the proportions of proteins and polysaccharides were high, and HLS were low in small granules, whilst the reverse was found in large granules (Fig. 1f; Supplementary Material Table S2).

### 3.2 Medium-sized granules have the highest methanogenic activity

Granules of all sizes were more methanogenically active when supplied with hydrogen than with the volatile fatty acids tested (Fig. 1e; Supplementary Material Table S3). Larger granules (Fractions H and I) were less active than smaller granules, regardless of substrate. Medium-sized granules (Fractions D–G) were generally the most active across all substrates tested.

### 3.3 Microbial community diversity decreases with increasing granule size

**Fig. 2:**
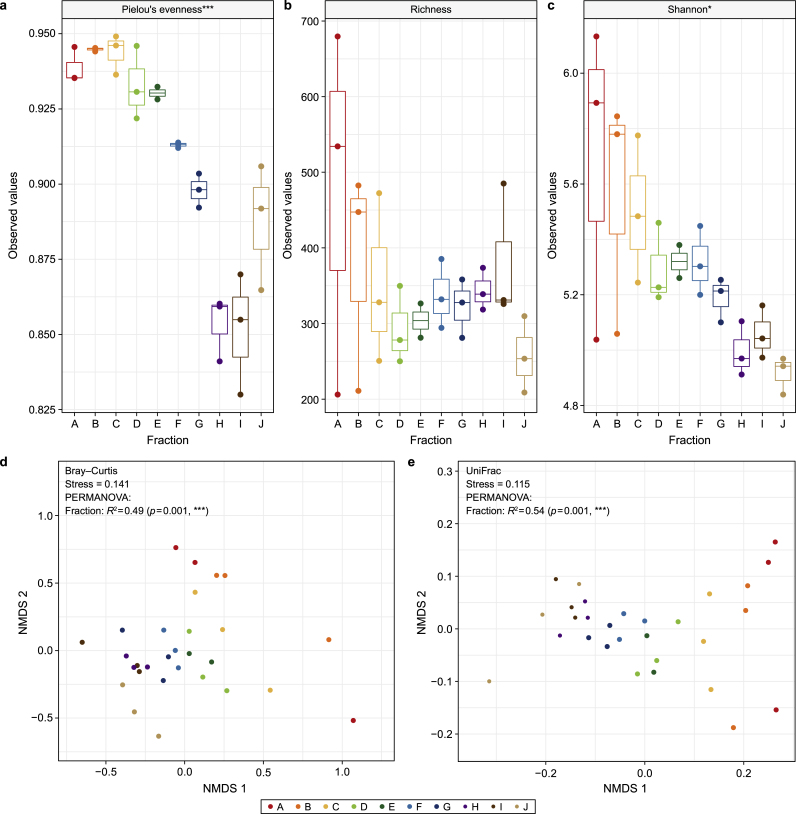
Microbial community diversity. Diversity was calculated from samples (*n* = 3) across the ten size fractions (A–J). **a**–**c**, Alpha diversity: box plot of the Pielou's evenness (**a**), rarefied richness (**b**), and Shannon Entropy (**c**). **d**–**e**, Beta diversity: Non-Metric Multidimensional Scaling (NMDS) using Bray-Curtis dissimilarity (**d**) and unweighted UniFrac distances (**e**), where each point corresponds to the community structure of one sample and size fractions are indicated by colours. Significance for panels **a**–**c** was determined using ANOVA and PERMANOVA for panels **d** and **e**, where ∗ (*p* < 0.05), ∗∗ (*p* < 0.01), or ∗∗∗ (*p* < 0.001).

Amplicon sequencing data analysis for the ten separate size fractions yielded a total of 3041 ASVs, with summary statistics of reads for *n* = 30 samples as follows: 1st Quartile: 12,270; Median: 16,385; Mean 16,132; 3rd Quartile: 18,936; and Max: 31,398. As granule size increased, significant reductions were observed in alpha diversity (Pielou's and Shannon's). Rarefied richness, however, did not change significantly across size (Fig. 2a–c). Beta diversity analysis, measured as both Bray Curtis and UniFrac, revealed significant (*p* = 0.001) clustering and gradients across the size fractions (Fig. 2d and e).

**Fig. 3:**
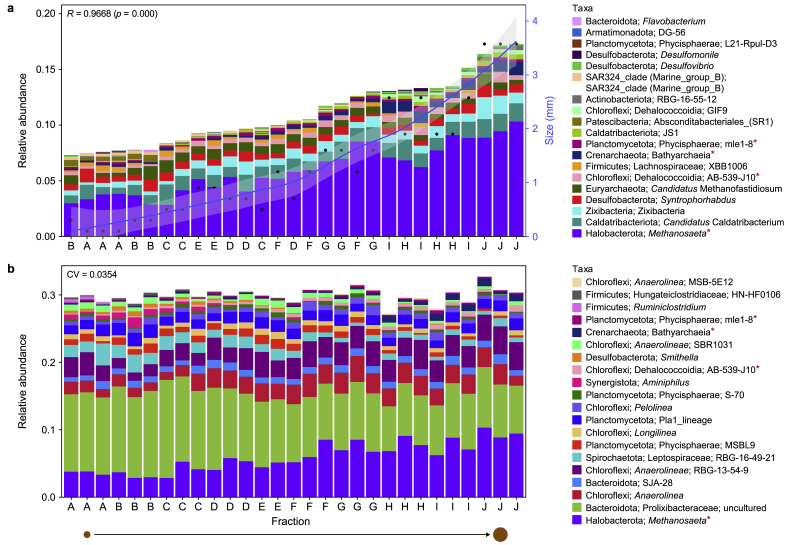
Dynamic and stable microbial community ensembles. Ensembles (a minimal subset of genera) returned after applying the ensemble quotient optimisation technique with size (mm) as a continuous predictor (**a**) and in uniform phenotypic variable mode (**b**). The bar plot in panel **a** represents the sorted relative abundance of the ensembles with a biplot of smooth size values in the top-left. The black dots represent the actual observed values of granule size (mm), whilst the blue line is a curve fitted through these values with the confidence interval drawn as shaded region around the curve. The bar plot in panel **b** represents the stable ensemble returned and shown as the relative abundance profiles with coefficient of variation (CV) values in the top-left. Low CV values indicate higher stability. Fitness value evolution graphs are available in Supplementary Materials. A red asterisk marks those genera returned in the stable and size-dependent ensembles.

The community diversity was significantly shifting across granule size, and EQO helped us further study the microbiome in the context of this environmental gradient. First, we identified a sub-community of 20 genera whose cumulative relative abundance correlated with size (Fig. 3a). In this instance, as size increased, not only did the cumulative relative abundances of the ensemble increase, but the relative abundances of many of the individual genera also increased. *Methanosaeta*, ‘*Candidatus* Caldatribacterium’, *Zixibacteria*, and *Bathyarchaeia* serve as examples. Next, EQO returned a stable subset of 20 genera, roughly 30 % of the total relative abundance (Fig. 3b). Whilst the individual abundances of those 20 genera may shift across size, this subset self-equilibrates, providing stability to the microbiome. Major members of this stable community included *Methanosaeta*, an uncultured member of the *Prolixibacteraceae,* and *Anaerolinea*.

### 3.4 Small granules harbour specific taxa; large granules are cosmopolitan

**Fig. 4:**
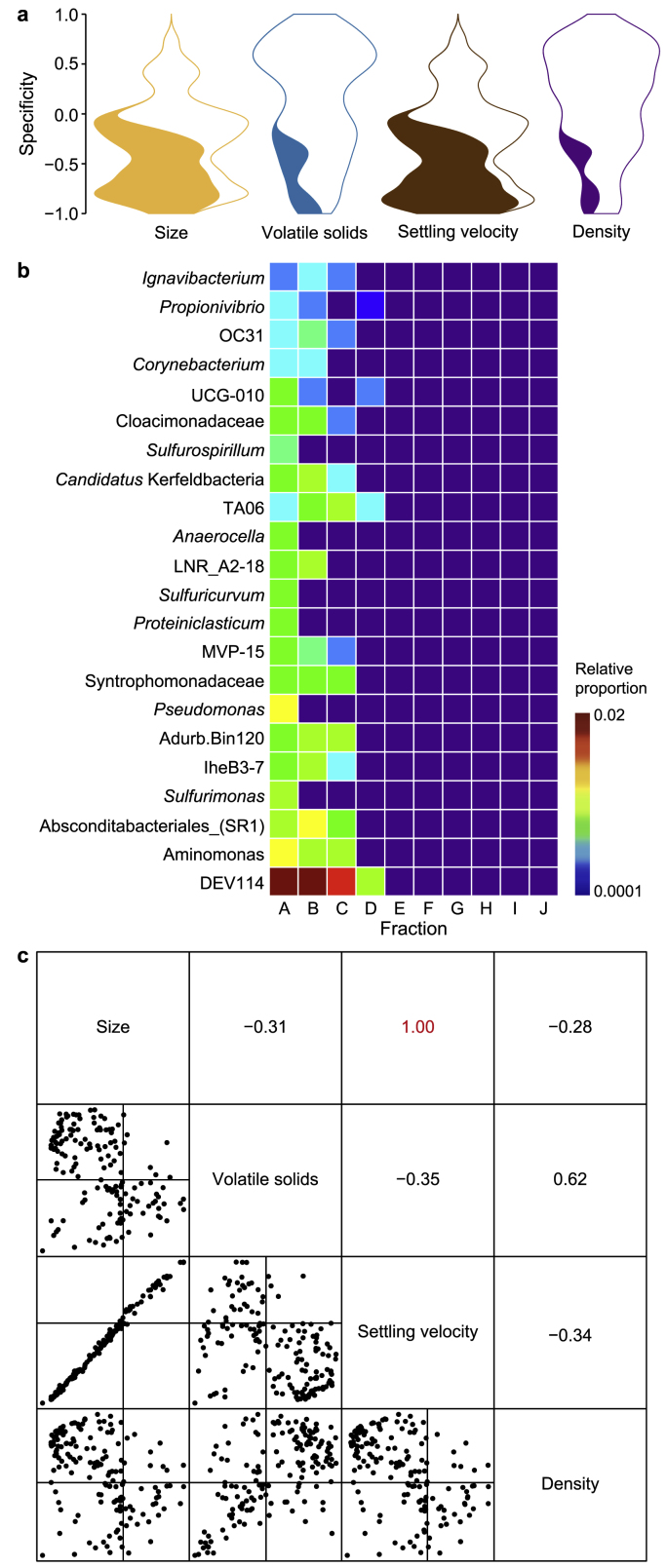
Specificity of taxa across size. **a**, Specificity values (Spec) shown as a violin plot with the area divided between genera with statistically significant specificity (dark) versus genera without (light) for the different environmental parameters. **b**, Heatmap of the relative proportions of the 22 genera identified as size-specific taxa. **c**, Pairwise Spec correlations with correlation coefficients (*R*) for each pairwise comparison of covariates are shown in this subplot's upper triangles for the data plotted in the lower triangle of this plot. Full taxa classifications and exact values are available (Supplemental Material Fig. S2).

Next, specificity (a null modelling tool) identified any genera existing within a narrow range of covariates (size, settling velocity, density, and volatile solids)—these taxa are considered ‘specific’ to that range. The null modelling approach calculated a ‘spec’ number, which offers a threshold to identify cosmopolitan taxa (>0) or specific (<0; Fig. 4a). Moreover, the algorithm identified the specific genera (22 in total), all specific to small granule sizes (Fig. 4b; Supplementary Material Fig. S2). Those 22 genera included many fermentative bacteria, utilising (amongst themselves) a wide range of substrates to produce a range of volatile fatty acids (VFA). The size-specific genera also included several unclassified organisms with yet unknown functions. The group of 22 represents a diverse range of phyla and likely contributes to the high diversity of the small granules. Medium and large granules, instead, contained more cosmopolitan microbial communities. Density and VS also contained a small number of specific taxa (Supplementary Materials Figs. S3 and S5). Pairwise comparisons of all covariates yielded correlation coefficients (*R*-values), which indicated a perfect correlation (or linear dependency) in specificity between size and settling velocity (Fig. 4c).

### 3.5 Granules contain both generalists and specialists

**Fig. 5:**
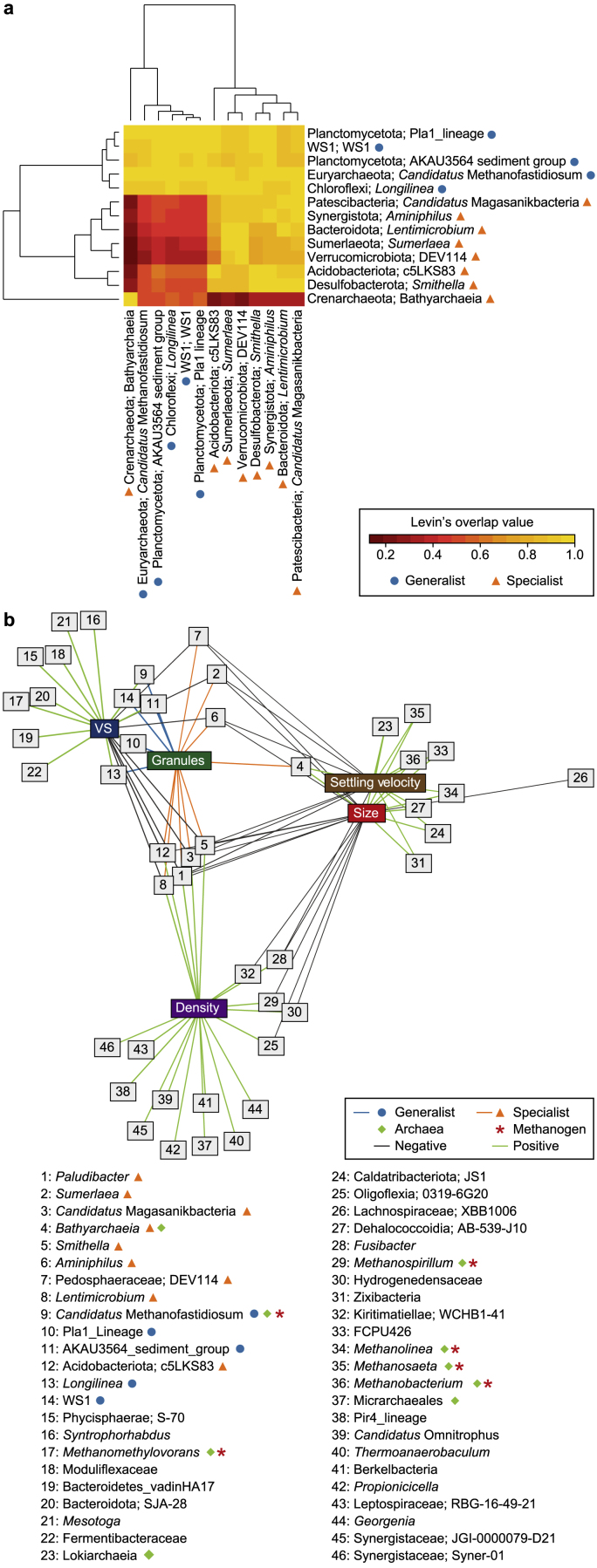
Generalists and specialists in anaerobic granules. Levin's ß_N_ identified generalists (light blue circles) and specialists (orange triangles) across all granule samples. These selected genera were used to calculate Levin's overlap (LO, **a**). Notably, the heatmap is not a symmetric map. LO_1,2_ and LO_2,1_ are not necessarily equal. When LO_1,2_ is equal to one, species 1 completely overlaps in all instances where species 2 exists. Conversely, when LO_2,1_ equals one, species 2 completely overlaps in all instances where species 1 exists. A network of relationships (**b**) was recovered after applying Levin's ß_N_ and Hurlbert's ß_N_ to find positive and negative associations concerning environmental properties measured in this study: size, density, settling velocity, and volatile solids (VS). Supporting data is available in Supplemental Materials (Supplementary Materials Figs. S6–S8).

Levin's ß_N_ identified five generalist and eight specialist genera across all samples (Fig. 5a). Most of the specialists were known fermenters. Interestingly, according to Hurlbert's ß_N_, nearly all generalists and specialists correlated negatively with size, settling velocity and VS (Fig. 5b). Several, however, showed positive correlations with density. *Bathyarchaeia* stands out as a specialist; this was the only genus with positive associations between size and settling velocity, and the observed Levin's overlap (LO) was very low with the other generalist and specialist genera. Notably, several archaea correlated positively with size, including *Bathyarchaeia*, *Lokiarchaeia*, and the methanogens *Methanolinea*, *Methanosaeta*, and *Methanobacterium*.

### 3.6 Granules become more methanogenic as they grow

**Fig. 6:**
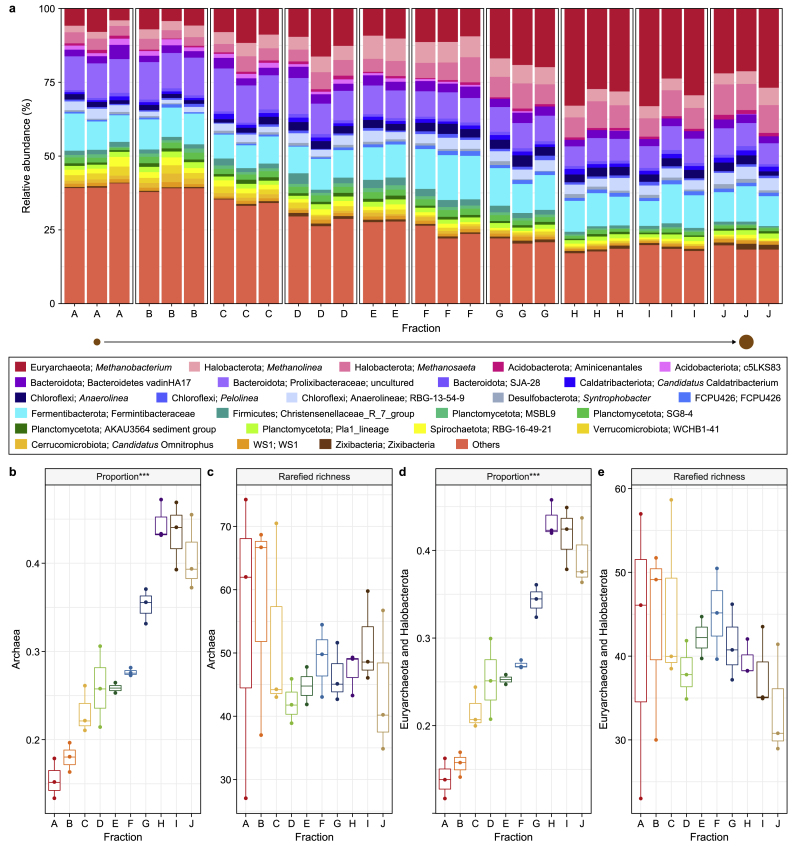
Diversity of archaeal groups. **a**, Microbial community structure is depicted as the relative abundance of the top-25 most abundant genera from triplicate samples across each size fraction, where ‘others’ refers to all genera not included in the ‘top-25’. **b**–**c**, Bar plots showing the proportion (**b**) and rarefied richness (**c**) of Archaea. **d**–**e**, The proportion (**d**) and rarefied (**e**) richness of the *Euryarchaeota* and *Halobacterota* groups across granule size. Significance for panels **b**–**e** was determined using ANOVA, where ∗ (*p* < 0.05), ∗∗ (*p* < 0.01), or ∗∗∗ (*p* < 0.001).

To further investigate and confirm the trend in archaeal abundances, we looked at the overall community structure and relative abundances of the 25 most abundant genera. Indeed, the three methanogenic archaea identified by Hurlbert's ß_N_ were the most abundant genera within the overall microbiome, and their relative abundances increased with increasing size (Fig. 6a). The proportions of archaea and the subgroup of methanogenic archaea (*Euryarchaeota* and *Halobacterota)* both significantly increased with increasing size (*p* < 0.001; Fig. 6b–d). However, the rarefied richness of these groups did not change significantly (Fig. 6c–e).

## 4 Discussion

### 4.1 Medium-sized granules may be the most optimal

One of the primary objectives of this study was to intensively characterise anaerobic granules from across a series of discrete sizes to identify patterns in biofilm development and potentially identify ‘prime’ biofilm size/age. We hypothesised that distinct granule sizes correspond to stages of biofilm development, in which small granules are ‘young’ and larger ones are ‘old’. Across each parameter explored, significant differences existed between granules of different sizes.

Volatile solids comprised a smaller proportion of the biofilms in fractions A–C. One of the key theories on granulation is the 'spaghetti theory', which proposes that during the initial stages of biofilm formation, cells attach to inorganic nuclei [40,41], which may have made up an inorganic core comprising a larger proportion of the solids in the smaller granules. As further cells attach and the biofilm grows, the organic fraction would become more important in larger granules, which is supported by the VS data presented. Notably, there is a significant drop in VS and density and a shift in LB-EPS between size fractions A and B. We believe that these changes point to an interesting shift in granule development. It is possible that between these sizes, we are observing the transition between a ‘pre-granule’ state and a more stable aggregate—a transition that warrants further study.

A distinct gradient was observed in the ultra-structural features across the size fractions. Small granules were flaky and undefined, whilst the largest granules were spherical and—in some cases—beginning to break apart. This was consistent with previous work that studied small, medium, and large granule sizes [17]. Granule size has been previously explained by environmental factors such as upflow velocity, hydrodynamics, and sludge bed fluidisation [42,43] and it is possible that these same factors also influence the changes in ultrastructure. Gradients in density and settleability profiles were also observed across size, whereby smaller granules were much denser than larger granules but had much lower settling velocities. Diaz et al. (2006) [24] also applied SEM using granules they had sliced in half, revealing cross-sections, observing that the largest granules had major cracks and void spaces that were less apparent in smaller granules. It is possible that such differences, or changes, in the structure of the granules also affect density: as granules become larger, they acquire more cracks, channels and void spaces from gas diffusion within the biofilm, which makes the biofilm less tightly packed and, as a result, less dense. Furthermore, previous studies have described stratification of the sludge bed in anaerobic digesters using granules, where larger granules occupy the bottom and smaller ones the top of the bed [[44], [45], [46]]—this is interesting if granules in a bioreactor are to be considered as a meta-community or meta-organism. More specifically, however, avoiding biomass wash-out is a key consideration in applying anaerobic granules in bioreactors, and the findings lead us to conclude that settleability, rather than density, is the driving force for stratification.

There appears to have been a clear, linear gradient characterised by reducing diversity and converging community structure across the size fractions, from small to large. This was somewhat counter to our initial assumption: diversity and rarefied richness would increase as the biofilm assembles and matures, especially as the biofilm simply contained significantly more cells. Both rarefied richness and diversity (measured by Shannon entropy) decreased significantly with granule size due to the gradual dominance of a sub-group of the ASVs. Interestingly, the dominant core group appeared to comprise three methanogenic archaea: *Methanosaeta*, *Methanolinea*, and *Methanobacterium,* which includes hydrogenotrophic methanogens and may explain the high methanogenic activity we measured against hydrogen as substrate. Indeed, granules may become more strictly anaerobic with increasing size, creating ideal conditions for expanding methanogenic populations.

Interestingly, medium-sized granules (Fractions D–G) contributed a volumetric majority to the biomass used for this experiment. Those were also the most methanogenically active granules and generally appeared to have the most ‘stable’ ultrastructure. This indicates that the medium-sized granules may be the most stable, least open to immigration, and most important for methane production. Moreover, the alpha diversity analysis showed that medium-sized granules were perhaps optimally diverse—containing a community rich in methane-producing archaea but not over-dominated by them. The digester system may self-regulate to select medium-sized granules as an optimal growth phase in which critical trophic groups are maintained, suggesting sophisticated ecological survival strategies. Furthermore, this may point to potential management strategies for digester operations where systems are managed to promote the emergence and existence of medium-sized granules.

### 4.2 Linking size with ecological patterns of the microbiome

In this study, we considered each granule size to be a different ecological ‘environment’ with its own unique properties. From this perspective, the dataset presented a unique opportunity to apply taxa-centric approaches to identify ecological roles and patterns within the microbiome and across size gradients. In particular, we could highlight, for the first time, the distinctive nature of the small granules (Fractions A–C). We found 22 rare genera specific to small granules and not elsewhere. Those taxa most likely contributed to the higher microbial diversity observed in smaller granules. Considering size as a proxy for biofilm age, we may speculate that as the young (small) granules form, they can attract and harbour a wide range of species. However, as the granule ages, grows, and continues to develop, rare taxa are out-competed by other, more robust taxa occupying the same metabolic niche.

Indeed, niche dynamics are a fundamental concept used to explain how environmental gradients and species interactions determine the abundance and/or activity of individual microorganisms within the community [47]. It has become increasingly popular to classify specific taxa as either generalists (microbes with a broad, non-discriminatory niche) or *specialists* (microbes with a narrow, discriminatory niche)[13,[48], [49], [50]]. Recently, it has been proposed that generalists—rather than elevated diversity, as has been commonly thought—provide resilience to a microbial community [50]. Here we identified five generalist and eight specialist genera. In general, the specialists displayed a very high co-occurrence or overlap (LO approaching or equal to 1) with one another, indicating that they are very unlikely to occupy the same niche space. A similar pattern was observed amongst the generalists. The exception was *Bathyarchaeia*, which (i) displayed a low co-occurrence with other generalists and specialists, (ii) was identified by EQO as a member of the stable and size-correlating sub-communities, and (iii) was the only specialist having a positive correlation with size and settling velocity. *Bathyarchaeia* are considered very metabolically diverse and one of the most abundant microorganisms on Earth—playing pivotal roles in global carbon cycling [51]. It remains unclear exactly which niche or ecological role the *Bathyarchaeia* are filling within anaerobic granules, but trends suggest that as granules age and grow, the *Bathyarchaeia* become more established.

### 4.3 Development model for anaerobic granules

The observations from this study—on gradients in ultrastructure, activity, EPS composition and community structure—culminate in the proposal of a development model for anaerobic granular biofilms. This model proposes that granules begin as very small, compact and structurally irregular, yet diverse, agglomerations of cells [15]. Such granules are considered to be ‘young’. As the biofilm ages, it grows into a medium-sized, highly active, structurally stable entity with a less diverse community structure, selecting rather for a community capable of efficient methane generation, i.e., a consortium completing the anaerobic digestion process without significant accumulation of intermediate by-products. We consider those granules to be ‘ripe’. Further ageing weakens the granule structure, forming cracks and voids [24]. Activity decreases, likely due to structural inefficiencies in the mass transport of substrates from one trophic group to another, but the diversity continues to converge primarily toward a methanogenic consortium. The granules may then be considered as ‘mature’. It is probable that the granules eventually break apart, but the small fragments are still comprised of an active consortium and form the basis for new ‘young’ granules [17,28]. The only observation that does not fully support this hypothesis is the set of observed gradients in rarefied richness and diversity—the linearity of the gradients does not necessarily indicate a circular trajectory. In particular, the source of the additional richness in Fraction A is unclear, but the surrounding medium (wastewater, in the case of bioreactors) will likely provide the necessary additional diversity to cultivate new young granules. Answers to these questions may require further experimentation, perhaps by following single granules within digesters over a growth cycle. Additionally, further characterisation of granule ecology and development (across sizes) could include (i) fluorescent *in situ* hybridisation (FISH) to study the spatial distribution of specific taxa or trophic groups as granules grow and (ii) metagenomics and/or metatranscriptomics to observe how gene expression and metabolic pathways develop within granular biofilms. Finally, based on commonalities across anaerobic granular sludge microbiomes [52], we hypothesise that the findings presented in this study would apply to all granular sludge sources; however, this should be confirmed.

### 4.4 Implications for bioreactor operation

In this study, we used size to profile the development of granules from a full-scale anaerobic digester. We observed several important ecological and physico-chemical trends across sizes. Medium-sized granules could likely be optimal for digester performance, and bioreactors may self-regulate or be regulated to optimise for medium sizes. Managing the upflow velocity has resulted in changes in the average granule size [42,53]. However, the size distribution often seems to self-select for medium sizes [17,28,53,54], regardless of the specific size range of a given digester. There is still a disconnect between performance metrics and microbial ecology regarding the day-to-day operation of full-scale environmental biotechnologies, such as anaerobic digesters. Here we show that something as simple as a size distribution of the biomass could yield a lot of information about the underlying biology. Indeed, movement up or down in average size, or overall size distribution, could indicate loss of activity or digester performance. It turns out size directly reflects the composition and potential activity of anaerobic granules and is easy to monitor.

## 5 Conclusions

Granule size has been an interesting variable since the discovery of granular sludge. However, this study is among the first to characterise granule size in such a highly-resolved way and to think about size in terms of granule development. We established connections between properties such as methanogenic activity, ultrastructure, density, extracellular polymeric substances, and ecological aspects while simultaneously modelling ecological interactions in relation to the specificity and niche preferences of the microbes. Moreover, this study is the first to suggest that, in the future, size could be an important on-site tool to make inferences about the biological ‘health’ of a digester. Our findings from this study could have profound implications for digester monitoring and be interesting from an ecological perspective concerning biofilm development.

Our study is unique as the study design entails using taxa-centric approaches (specificity and niche-breadth analysis) to establish distinct ecological roles of microorganisms. This is only possible if the environment and the associated environmental properties (size, VS, settling velocity, and density) are explicitly considered in deriving the analytical approaches that reveal underlying ecological phenomena. This viewpoint differs from traditional study-centric approaches, where a few size fractions have been considered without tying them to the underlying ecology. A growing literature suggests that the proportional representation of generalist and specialist species in an ecosystem (and not necessarily the total diversity of the ecosystem) have important roles in ecological stability. Therefore, exploring these ecological roles of different microorganisms in the context of granule size and development may complete the gaps in our understanding, which has not been possible in previous studies.

In summary, ecophysiolological and physico-chemical gradients were apparent in anaerobic granules across the highly-resolved set of size fractions investigated, indicating that aggregate size matters for structure and function. In general, medium-sized granules showed greater methanogenic activities and had a more ‘granular’ ultrastructure. Furthermore, it appeared that, as such biofilms developed, the microbial community significantly lost diversity and became dominated by a small number of highly abundant methanogenic archaea. The microbial communities of small granules contained 22 genera, which were highly specific to the small granule sizes, while the microbial communities of medium and larger granules were more cosmopolitan. Additionally, we identified five generalists and eight specialists within the granule communities and suggested that the presence of generalists may provide stability and resilience to the microbiome as granules grow. The unique combination of physico-chemical and ecological data from this study suggests that medium-sized granules may be optimal in terms of structure and function, and granules may follow a series of biofilm development stages that self-select for mostly medium-sized granules in a bioreactor that also includes smaller and larger aggregates. Indeed, operating a digester toward further selection for medium-sized granules might result in optimally efficient conversions and bioenergy production.

## CRediT authorship contribution statement

**Anna Trego:** Writing – original draft, Visualization, Methodology, Investigation, Funding acquisition, Formal analysis, Data curation, Conceptualization. **Cristina Morabito:** Data curation. **Isabelle Bourven:** Supervision, Resources, Methodology, Investigation, Data curation. **Giles Guibaud:** Supervision, Resources. **Vincent O'Flaherty:** Writing – review & editing, Supervision, Resources, Project administration, Funding acquisition. **Gavin Collins:** Writing – review & editing, Visualization, Supervision, Resources, Project administration, Methodology, Funding acquisition, Conceptualization. **Umer Zeeshan Ijaz:** Writing – review & editing, Visualization, Supervision, Software, Resources, Project administration, Methodology, Investigation, Funding acquisition, Formal analysis, Conceptualization.

## Declaration of competing interest

The authors declare that they have no known competing financial interests or personal relationships that could have appeared to influence the work reported in this paper.
