# Supplementary material for: Size matters: Anaerobic granules exhibit distinct ecological and physico-chemical gradients across biofilm size
Source: Environ Sci Ecotechnol. 2025 Mar 27;25:100561. doi: 10.1016/j.ese.2025.100561 (PMC12003022; doi:10.1016/j.ese.2025.100561)
Supplement: Multimedia component 1 [file mmc1.docx]

**SIZE MATTERS: ANAEROBIC GRANULES EXHIBIT DISTINCT ECOLOGICAL AND PHYSICO-CHEMICAL GRADIENTS ACROSS BIOFILM SIZE**

**Trego et al 2025**

**Supplementary Information**

*1. Bioinformatics*

Abundance tables were generated by constructing amplicon sequencing variants (ASVs) using the Qiime2 pipeline and the DADA2 algorithm (Bolyen et al., 2019) with details given at [<https://github.com/umerijaz/tutorials/blob/master/qiime2_tutorial.md>]. Within the workflow, qiime feature-classifier was used to classify the ASVs against SILVA SSU Ref NR database release v.138, and then qiime phylogeny align-to-tree-mafft-fasttree generated the rooted phylogenetic tree. The biom file for the ASVs was generated by combining the abundance table with taxonomy information using biom utility available in qiime2 workflow. In addition, we have removed contaminants such as chloroplasts and mitochondria as is recommended in taxonomy-based filtering of artifacts in the Qiime2 workflow (<https://docs.qiime2.org/2021.2/tutorials/filtering/>).

*2. Statistical analyses*

**2.1 Diversity measurements**

The vegan package (Oksanen et al., 2015) was used for alpha and beta diversity analyses. For alpha diversity measures we used: **(1)** *rarefied richness* – the estimated number of species/features in a rarefied sample (to minimum library size); **(2)** *Shannon entropy* – a commonly used index to measure balance within a community; and **(3)** *Pilou’s evenness,* which compares the actual diversity values to the maximum possible diversity value, and is constrained between 0 and 1.0, whereby lower values will indicate more variation in abundance between different ASVs in the community. Non-metric multidimensional scaling (NMDS) plots of ASVs using three different distance measures were made using Vegan’s metamds() function: **(1)** *Bray-Curtis,* which is a distance metric that considers only ASV abundance counts; **(2)** *Unweighted Unifrac,* which is a phylogenetic distance metric that calculates the distance between samples by taking the proportion of the sum of unshared branch lengths in the sum of all the branch lengths of the phylogenetic tree for the ASVs observed in two samples, and without taking into account their abundances; and **(3)** *Weighted Unifrac,* which is a phylogenetic distance metric combining phylogenetic distance with relative abundances. This places emphasis on dominant ASVs or taxa. Unifrac distances were calculated using the phyloseq package (McMurdie and Holmes, 2013).

Analysis of variance (ANOVA) was performed using Vegan’s Adonis() against distance matrices (Bray-Curtis/Unweighted Unifrac/Weighted Unifrac). This function, referred to as PERMANOVA, fits linear models to distance matrices and used a permutation test with pseudo-F ratios.

**2.2 Ensemble Quotient Optimization**

To identify a minimal subset of species that either remain stable across all conditions, or change according to environmental covariates, we incorporated the Ensemble Quotient Optimization (EQO) approach (Shan et al., 2023). The approach uses a relative abundance table, called community matrix $\boldsymbol{M}$ (*P* ASVs over *n* samples), where the goal is to obtain a vector $\boldsymbol{x}\in\left( 0,1 \right)^{P}$ where the *i^th^* position in the vector is either 0 or 1, i.e., a subset of species with values 1 belong to an ensemble which we are interested in recovering. This ensemble is recovered in the context of a phenotype/predictor variable $\boldsymbol{y}$ by optimizing an *Ensemble Quotient* $EQ=\frac{\boldsymbol{x}^{T}\boldsymbol{Qx}}{\boldsymbol{x}^{T}\boldsymbol{Px}}$, through a genetic algorithm (an optimization algorithm), where $\boldsymbol{P}$ and $\boldsymbol{Q}$ are algebraic transformations of the community matrix that capture the covariance between species, and the covariance between species and $\boldsymbol{y}$. The choice of $\boldsymbol{y}$ dictates what ensemble we recover, and can be used in two cases: a) If the interest lies in an ensemble of species that remain stable for a set of samples, then $\boldsymbol{y}$ is considered uniform i.e., consisting of 1s, with $\boldsymbol{Q=}\boldsymbol{M}^{T}\boldsymbol{1}\boldsymbol{1}^{T}\boldsymbol{M}$, and $\boldsymbol{P=}\boldsymbol{M}^{T}\boldsymbol{M-}\frac{2}{n}\boldsymbol{M}^{T}\boldsymbol{1}\boldsymbol{1}^{T}\boldsymbol{M+}\frac{\boldsymbol{1}}{n^{2}}\boldsymbol{M}^{T}\boldsymbol{1}\boldsymbol{1}^{T}\boldsymbol{1}\boldsymbol{1}^{T}\boldsymbol{M}$**;** b) If the interest lies in an ensemble of species whose cumulative abundance correlates with a continuous physico-chemical parameter y, then we optimize the algorithm with $\boldsymbol{Q=}\boldsymbol{M}_{0}^{T}\boldsymbol{y}_{0}\boldsymbol{y}_{0}^{T}\boldsymbol{M}_{0}$, $\boldsymbol{P=}\boldsymbol{M}_{0}^{T}\boldsymbol{M}_{0}$ ($\boldsymbol{M}_{0}$ is the centered community matrix $\boldsymbol{M}$ whose column means are zero with $\boldsymbol{y}_{0}$ also a centered version of $\boldsymbol{y}$). Within the context sizes, we have used the case (a) to see which subset of microbes do not change over the whole range of sizes (quality of fit is returned as Coefficient of Variation CV), whilst, case (b) was used to see which subset of microbes have a relationship with the changes in size (quality of fit is returned as a correlation coefficient between the continuous outcome and the cumulative abundance of the ensemble). To optimize the EQ to obtain $\boldsymbol{x}$, we followed the genetic algorithm optimization located at <https://github.com/Xiaoyu2425/Ensemble-Quotient-Optimization>. In the genetic algorithm, we have used the following parameterizations: a population size of 100 solutions, maximum of 600 generations, and a maximum 20 taxa (ASVs collated at genus level) to be returned as an ensemble.

**2.3 Microbial Niche Breadth, Overlap and Specificity**

To identify the roles of microbes within the context granule sizes, we have used the R’s MicroNiche package (Finn et al., 2020). The aim is to identify generalist (that should exist in majority of the pens) and specialist (that should exist in some pens) microbial species as well as size-dependent positive/negative association of microbial species with covariates such as size, settling velocity, density and volatile solids concentrations.

Before, applying these approaches, we filtered out genera using the limit of quantification (LOQ) approach as per author’s instruction. Briefly, LOQ filters out microbes that fall below a “decision boundary”, calculated from the distribution of microbes with 95% certainty that these microbes will fall within a null distribution where the mean microbial abundance is zero. To calculate the standard deviation of the null distribution, the lognormal rank distribution of the microbes with the dataset was fitted with $S\left( R \right)=S_{0}e^{-a^{2}R^{2}}$ where log abundance of microbe $S$ at rank $R$ is dependent on coefficient $a$ and rank $R$ calculated as $a=\sqrt{\frac{\ln S_{0}}{S_{m}}/R^{2}}$ where $S_{m}$ is the lowest taxon abundance of $S$. To calculate LOQ, we fit the above log normal model to data, and LOQ is then determined as the overlap between the null hypothesis (i.e., a microbe’s mean abundance is zero) and where the microbe falls within 1 standard deviation of the above model.

After filtering out the genera, we then calculated the niche breadth as Levins’ $B_{N}=\frac{1}{R}\sum_{i=1} p_{i}^{2}$, where $p_{i}$ is the proportional abundance of a genus in the $i$-th size, with total number of sizes (environments) being $R$ (10 in this case). If $B_{N}$ approaches 1 for a given genus, then it is considered as a “generalist”, whilst if it approaches $1/R$, then it can be tagged as a “specialist”. To derive the p-value for Levins’ $B_{N}$ i.e., if it we can call a genus a generalist or a specialist with great certainty, a null modelling approach is used, where a random normal distribution of 999 possible niche breadths were produced for a genus, and allows a p-value to be assigned depending on whether a genus’s $B_{N}$ is greater or lower than the mean of the null model. As per author’s recommendation, after applying null modelling, the 5^th^ Quantile and 95^th^ Quantile were obtained to tag the genera as specialist if its $B_{N}$ < 5^th^ Quantile, and generalist, if its $B_{N}$ > 95^th^ Quantile. Those that fell in the inter-range were tagged as undecided.

In the second step, we then calculated the overlap of these undecided/specialist/generalists using Levins’ Overlap formula $LO_{i,j}=\frac{\sum_{i,j=1} (p_{ir})(p_{jr})}{\sum_{i=1} (p_{ir}^{2})}$, where $p_{i}$ is the proportional abundance of genus $i$ in the $r$-th pen, and $p_{j}$ is the abundance of genus $j$ in the $r$-th pen, where $i$ and $j$ were selected after tagging an individual genus as undecided, specialist or generalist.

In addition to Levins’ $B_{N}$, we also calculated Hurlbert’s $B_{N}=\frac{1}{\sum_{i=1} \frac{p_{i}^{2}}{r_{i}}}$, where we have an additional $r_{i}$ proportional covariate data (*size, settling velocity, density and volatile solids concentrations*) in the formula. The model yields a value between 0 and 1 for each genus and corresponding covariate, indicating whether there is an inverse (~0) or a positive relationship (~1), with 0.5 indicating no relationship to the covariate. Similar to Levins’ $B_{N}$ approach, a null modelling procedure was considered by generating a random normal distribution of 999 possible niche breadth, and by tagging it as “negative” if its $B_{N}$ < 5^th^ Quantile, and “positive”, if its $B_{N}$ > 95^th^ Quantile. To determine positive and negative relationship (potentially symbiosis and antagonism) between the genera, we have used Proportional Overlap formula $PO_{i,j}=1-\left( \frac{X\cap Y}{X\cup Y} \right)$, where *X* (for genus *i*) and *Y* (for genus *j*) are the Feinsinger’s PS, calculated as $\mathrm{PS}=1-0.5\sum_{i=1} |p_{i}-r_{i}|$, and is similar to Hurlbert’s $B_{N}$. The Proportional Overlap $PO_{i,j}$ is a Jaccard similarity coefficient which approaches 0 for genus pairs that are inversely related to each other and approaches 1 for genus pairs that are positively related to each other. Note that we have calculated the $PO_{i,j}$ for those genera that were identified as undecided/positive/negative after applying Hurlbert’s $B_{N}$.

Next, we wanted to explore if certain genera exist within a narrow range of covariates considered in this study (*size, settling velocity, density and volatiles solids concentrations*). This is particularly important on the basis of granular growth, as age could potentially select for certain genera. For this purpose we have used R’s Specificity Package (Darcy et al., 2022) that calculates Rao’s Quadratic Entropy (RQE) as $RQE=\sum_{i=1}^{s-1} \sum_{j=i+1}^{s} D_{ij}p_{i}p_{j}$ where genus abundance $p_{i}p_{j}$ is the multiplication of the abundance of a specific genus in samples $i$ and $j$, respectively, weighted by the difference in the covariate value (*size, settling velocity, density and volatiles solids concentrations*) $D_{ij}$. A null modelling procedure is then applied (statistical effect size) where 999 random permutations were obtained for the abundance table, and RQE values were then obtained for these random permutations. Deviation of the original RQE from the average of RQEs of these random permutations then gives a “Spec” number, ranging from -1 to +1, with 0 as the null hypothesis that the genus weights are randomly ordered with regard to sample identity, with perfect *specificity* when Spec approaches -1 and perfect *cosmopolitanism* when spec approaches +1, and with the null modelling procedure providing additional p-values for significance. For visualization purposes, we have only plotted the lowest 25^th^ quartile (i.e., those genera that were specific).

**Supplemental Results**

**Table S1.** Percent by volume, percent VS, average settling velocity of granules across size fractions

|  |  | **Volatile Solids (% of TS)** | **Settling Velocity (m/s)** | |
| --- | --- | --- | --- | --- |
| Fraction | **Volume (%)** | Average | Average | STD |
| A | 3.53 | 86.27 | 0.00 | 0.00 |
| B | 8.33 | 70.46 | 0.00 | 0.00 |
| C | 6.06 | 89.05 | 0.00 | 0.00 |
| D | 10.60 | 92.16 | 0.01 | 0.00 |
| E | 20.70 | 92.67 | 0.01 | 0.00 |
| F | 33.07 | 92.45 | 0.01 | 0.00 |
| G | 11.87 | 92.20 | 0.02 | 0.00 |
| H | 0.50 | 91.14 | 0.02 | 0.00 |
| I | 5.30 | 91.57 | 0.03 | 0.00 |
| J | 0.03 | 90.12 | 0.04 |  |

**Table S2.** Percent composition of loosely bound and tightly bound EPS

|  | **Loosely bound EPS (% of total EPS)** | | | **Tightly bound EPS (% of total EPS)** | | |
| --- | --- | --- | --- | --- | --- | --- |
| Fraction | Proteins | Humic-like Substances | Polysaccharides | Proteins | Humic-like Substances | Polysaccharides |
| A | 55.61 | 7.87 | 36.52 | 55.14 | 18.07 | 26.80 |
| B | 20.07 | 35.22 | 44.70 | 43.83 | 29.30 | 26.87 |
| C | 36.00 | 42.39 | 21.62 | 64.13 | 14.16 | 21.72 |
| D | 19.09 | 68.68 | 12.23 | 57.50 | 17.32 | 25.19 |
| E | -0.31 | 89.10 | 11.21 | 54.95 | 17.76 | 27.29 |
| F | 5.20 | 82.58 | 12.22 | 53.40 | 26.66 | 19.94 |
| G | 17.30 | 58.82 | 23.88 | 59.85 | 17.53 | 22.63 |
| H | 9.36 | 78.28 | 12.35 | 46.24 | 33.61 | 20.15 |
| I | 10.18 | 84.65 | 5.17 | 44.14 | 32.65 | 23.21 |

**Table S3.** Average SMA values across size fractions

|  | **Acetate SMA** | | **Propionate SMA** | | **Butyrate SMA** | | **Hydrogen SMA** | |
| --- | --- | --- | --- | --- | --- | --- | --- | --- |
| Fraction | Average | STD | Average | STD | Average | STD | Average | STD |
| A | 57.32 | 9.15 | 0.94 | 0.79 | 37.12 | 17.39 | 279.41 | 55.09 |
| B | 212.00 | 16.73 | 66.58 | 1.89 | 84.80 | 13.16 | 521.28 | 90.78 |
| C | 106.23 | 2.89 | 59.75 | 15.54 | 27.12 | 22.93 | 265.30 | 51.47 |
| D | 138.53 | 17.29 | 96.15 | 28.57 | 26.36 | 12.09 | 136.51 | 23.62 |
| E | 278.38 | 40.79 | 79.84 | 21.10 | 74.34 | 14.52 | 449.32 | 57.21 |
| F | 263.85 | 69.42 | 100.52 | 27.29 | 139.25 | 55.53 | 293.47 | 15.51 |
| G | 318.19 | 31.27 | 82.58 | 13.57 | 53.97 | 7.12 | 408.97 | 28.91 |
| H | 70.46 | 0.54 | 40.36 | 4.17 | 1.41 | 1.17 | 162.76 | 27.80 |
| I | 80.02 | 27.88 | 27.32 | 3.60 | 0.08 | 0.21 | 125.84 | 13.93 |

**Fig S1.** Granule ultrastructure from representative granules across the ten size fractions (A-J) imaged using scanning electron microscopy (SEM).

**Fig S2.** For the genera identified as “Specific” in Figure 6 for *Size*, the profile of the genera in lowest 25^th^ quartile of *Spec* values are shown.

**Fig S3.** For the genera identified as “Specific” in Figure 6 for *Density*, the profile of the genera in lowest 25^th^ quartile of *Spec* values are shown.

**Fig S4.** For the genera identified as “Specific” in Figure 6 for *Settling velocity*, the profile of the genera in lowest 25^th^ quartile of *Spec* values are shown.

**Fig S5.** For the genera identified as “Specific” in Figure 6 for *VS*, the profile of the genera in lowest 25^th^ quartile of *Spec* values are shown.

**Fig S6.** Supporting information for Main Manuscript Figure 5. In **(A)** we show the rank distribution of the taxa observed (black), the lognormal rank distribution (red), and the limit of quantification threshold (blue). Any taxa that fall below the limit of quantification were excluded from the analyses. The null model distributions **(B)** generated from applying Levin’s ß_N_ calculated from 999 randomly generated taxon distributions. Red dotted lines indicate the fifth and 95th quantiles.

**Fig S7.** Further analysis for results shown in main manuscript Fig 5. For each analysis, the left figures show the rank distribution of the taxa observed in the dataset (black circles), the lognormal rank distribution model (red circles) and the limit of quantification threshold (blue circles) is 1 standard deviations from zero. Any taxa that fall below the limit of quantification were excluded from the analyses. The right figure represents the null model distributions generated from applying Hurlbert's B_N_ calculated from 999 randomly generated taxon distributions. Red dotted lines indicate the fifth and 95th quantiles. Taxa that are high when an environmental property (***A.*** *Density,* ***B****. Settling Velocity,* ***C****. Size, or* ***D****. VS*) is low have a Hurlbert's B_N_ below the 5th quantile, and conversely, taxa that are high when the environmental property is high have a Hurlbert's B_N_ above the 95th quantile of those null models.

**Fig S8.** The Proportional Overlap $PO_{i,j}$ which approaches 0 for genus pairs that are inversely related to each other, and approaches 1 for genus pairs that are positively related to each other.

*References*

Bolyen, E., Rideout, J.R., Dillon, M.R., Bokulich, N.A., Abnet, C.C., Al-Ghalith, G.A., Alexander, H., Alm, E.J., Arumugam, M., Asnicar, F., 2019. Reproducible, interactive, scalable and extensible microbiome data science using QIIME 2. Nat. Biotechnol. 37, 852–857.

Darcy, J.L., Amend, A.S., Swift, S.O.I., Sommers, P.S., Lozupone, C.A., 2022. specificity: an R package for analysis of feature specificity to environmental and higher dimensional variables, applied to microbiome species data. bioRxiv 2021.11.06.467582. https://doi.org/10.1101/2021.11.06.467582

Finn, D.R., Yu, J., Ilhan, Z.E., Fernandes, V.M.C., Penton, C.R., Krajmalnik-Brown, R., Garcia-Pichel, F., Vogel, T.M., 2020. MicroNiche: an R package for assessing microbial niche breadth and overlap from amplicon sequencing data. FEMS Microbiol. Ecol. 96, fiaa131. https://doi.org/10.1093/femsec/fiaa131

McMurdie, P.J., Holmes, S., 2013. phyloseq: An R Package for Reproducible Interactive Analysis and Graphics of Microbiome Census Data. PLoS One 8, e61217.

Oksanen, J., Blanchet, F., Kindt, R., Legendre, P., Minchin, P.R., O’hara, R., Simpson, G.L., Solymos, P., Stevens, H.H., Wagner, H., 2015. Vegan: community ecology package. R Package version 2.2-1.

Shan, X., Goyal, A., Gregor, R., Cordero, O.X., 2023. Annotation-free discovery of functional groups in microbial communities. Nat. Ecol. Evol. 7, 716–724. https://doi.org/10.1038/s41559-023-02021-z
